# Supplementary material for: Rapid Profiling of Protein Complex Reorganization in Perturbed Systems
Source: J Proteome Res. 2023 Apr 14;22(5):1520–36. doi: 10.1021/acs.jproteome.3c00125 (PMC10167687; doi:10.1021/acs.jproteome.3c00125)
Supplement: Supplementary file 1 — pr3c00125_si_001.pdf [file pr3c00125_si_001.pdf]

Supporting information for:

Rapid profiling of protein complex re-organization in perturbed systems

Authors

Isabell Bludau[1,2#], Charlotte Nicod[1#], Claudia Martelli[1,3#], Peng Xue[1], Moritz Heusel[1,4], Andrea Fossati[1,5,6,7], Federico Uliana[1,8], Fabian Frommelt[1], Ruedi Aebersold[1], Ben C. Collins[1,9*]

[1]Department of Biology, Institute of Molecular Systems Biology, ETH Zürich, Zürich, Switzerland 8092

[2]Department of Proteomics and Signal Transduction, Max Planck Institute of Biochemistry, Martinsried, Germany 82152

[3]Present address: Bruker Daltonics, 8117 Fällanden, Switzerland

[4]Division of Infection Medicine (BMC), Department of Clinical Sciences, Lund University, Lund, Sweden 22184

[5]University of California San Francisco, Quantitative Biosciences Institute (QBI), San Francisco, CA, 94158, USA

[6]University of California San Francisco, Department of Cellular and Molecular Pharmacology, San Francisco, CA, 94158, USA

[7]J. David Gladstone Institutes, San Francisco, CA 94158, USA

[8]Department of Biology, Institute of Biochemistry, ETH Zürich, Zürich, Switzerland 8052

[9]School of Biological Sciences, Queen's University of Belfast, 19 Chlorine Gardens, Belfast, BT9 5DL, UK

[#]equal contribution

*Correspondence to: ben.collins@qub.ac.uk

Contents:

Figure S1 Effect of chromatographic optimization on peptide detection rates
Figure S2 Effect of DIA/SWATH optimization on peptide detection rates
Figure S3 SEC molecular weight calibration
Figure S4 Peptide precursor and protein numbers detected by SEC fraction
Figure S5 Pairwise SEC alignment
Figure S6 Cyclic loess normalization
Figure S7 Peptide detection rates and effect of filtering
Figure S8 Proteins detected in assembled vs. monomeric state
Figure S9 Detected protein feature summary
Figure S10 Protein mass in assembled state
Figure S11 Protein complex detection rates as a fraction of complex hypotheses
Figure S12 Distribution of SEC protein features dependent on MW and detection in complex-centric analysis

Supplementary Figures

A

Condition	Column length (cm)	Flow rate (nl/min)	Gradient lenght (min)	N variable windows	MS² scan time (msec)	MS² mass range	Sample load (µgs)
A) Standard DIA/SWATH method	20	300	90	64	50	50-2000	1
B) Shorten column	10	300	90	64	50	50-2000	1
C) Increase flowrate	10	1000	90	64	50	50-2000	1
D) Short gradient-Method 1	10	1000	20	64	25	50-2000	1
E) Short gradient-Method 2	10	1000	20	32	50	50-2000	1
F) Short gradient-Method 3	10	1000	20	100	15	50-2000	1
G) Short gradient-Method 4	10	1000	20	64	25	350-1500	1
H) Short gradient-Method 5	10	1000	20	64	25	50-2000	2

B

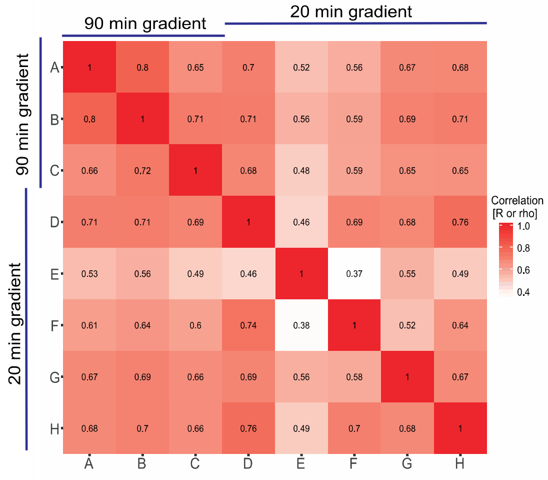

C

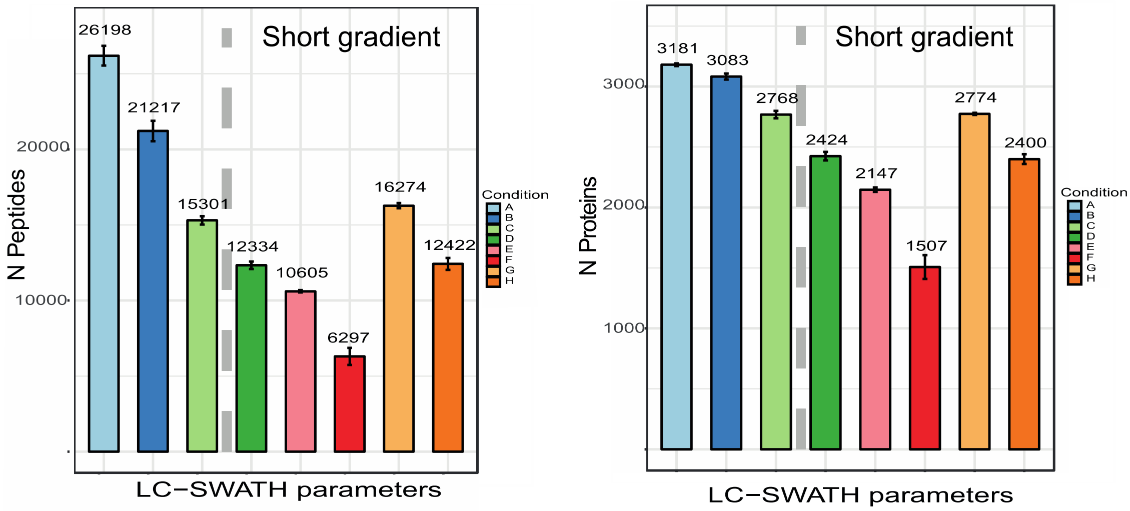

Supplementary Figure 1 – Effect of chromatographic optimization on peptide detection rates

Effect of the column length, flow rate, gradient length and number of DIA/SWATH variable windows in the DIA/SWATH performances (sample correlation, number of peptides and proteins identification). All the

runs were acquired with Eksigent nanoLC Ultra AS2 1D Plus and expert 400 autosampler system (Eksigent, Dublin, CA) coupled to a TripleTOF 5600 (Sciex) through a NanoSpray III ion source (Sciex). The 20 cm and 10 cm nanoLC columns were packed in house using a 75 µm inner diameter PicoFrit emitter (New Objective, Woburn) and ProntoSIL 3 um, 200 Å particles (Bischoff Chromatography, Germany).

For the chromatographic separation an aqueous solution with 2% ACN/0.1 FA % was used as buffer A, while a 98 % ACN%/0.1% FA solution as buffer B.

Panel A. Short scheme of the different conditions tested.

A) Standard DIA/SWATH method using a scheme of 64 variably sized precursor co-isolation windows. The SWATH windows cover the precursor ions in the range of 360-1460 m/z and 50-2000 in the MS2 SWATH scans, the accumulation time was 250 ms for the MS1 and 50 ms for each SWATH window, resulting in a cycle time of 3.5 s. For fragmentation, it was applied a rolling collisional energy with a collisional energy spread of 15 eV. 1 µg of peptides were loaded in the sample loop. The separation was carried out with a 20-cm capillary column at 300 nl/min flow for a 90-min gradient from 5 to 30% of buffer B.

B) Shorten column using a scheme of 64 variably sized precursor co-isolation windows. The SWATH windows cover the precursors ions in the range of 360-1460 m/z and 50-2000 in the MS2 SWATH scans, the accumulation time was 250 ms for the MS1 and 50 ms for each SWATH window, resulting in a cycle time of 3.5 s. For fragmentation, it was applied a rolling collisional energy with a collisional energy spread of 15 eV. 1 µg of peptides were loaded in the sample loop. The separation was carried out with a 10-cm capillary column at 300 nl/min flow for a 90-min gradient from 5 to 30% of buffer B.

C) Increased separation flowrate using a scheme of 64 variably sized precursor co-isolation windows. The SWATH windows cover the precursors ions in the range of 360-1460 m/z and 50-2000 in the MS2 SWATH scans, the accumulation time was 250 ms for the MS1 and 50 ms for each SWATH window, resulting in a cycle time of 3.5 s. For fragmentation, it was applied a rolling collisional energy with a collisional energy spread of 15 eV. 1 µg of peptides were loaded in the sample loop. The separation was carried out with a 10-cm capillary column at 1000 nl/min flow for a 90-min gradient from 5 to 30% of buffer B.

D) Short gradient-Method 1. 64 variably sized precursor co-isolation windows scheme. The SWATH windows cover the precursors ions in the range of 360-1460 m/z and 50-2000 in the MS2 SWATH scans, the accumulation time was 250 ms for the MS1 and 25 ms for each SWATH window, resulting in a cycle time of 1.9 s. For fragmentation, it was applied a rolling collisional energy with a collisional energy spread of 15 eV. 1 µg of peptides were loaded in the sample loop. The separation was carried out with a 10-cm capillary column at 1000 nl/min flow for a 20-min gradient from 5 to 30% of buffer B.

E) Short gradient-Method 2. 32 variably sized precursor co-isolation windows scheme. The SWATH windows cover the precursors ions in the range of 360-1460 m/z and 50-2000 in the MS2 SWATH scans, the accumulation time was 250 ms for the MS1 and 50 ms for each SWATH window, resulting in a cycle time of 1.9 s. For fragmentation, it was applied a rolling collisional energy with a collisional energy spread of 15 eV. 1 µg of peptides were loaded in the sample loop. The separation was carried out with a 10-cm capillary column at 1000 nl/min flow for a 20-min gradient from 5 to 30% of buffer B.

F) Short gradient-Method 3. 100 variably sized precursor co-isolation windows scheme. The SWATH windows cover the precursors ions in the range of 360-1460 m/z and 50-2000 in the MS2 SWATH scans, the accumulation time was 250 ms for the MS1 and 15 ms for each SWATH window, resulting in a cycle time of 1.8 s. For fragmentation, it was applied a rolling collisional energy with a collisional energy spread of 15 eV. 1 µg of peptides were loaded in the sample loop. The separation was carried out with a 10-cm capillary column at 1000 nl/min flow for a 20-min gradient from 5 to 30% of buffer B.

G) Short gradient-Method 4. 64 variably sized precursor co-isolation windows scheme. The SWATH windows cover the precursors ions in the range of 360-1460 m/z and 350-1500 in the MS2 SWATH scans, the accumulation time was 250 ms for the MS1 and 25 ms for each SWATH window, resulting in a cycle time of 1.9 s. For fragmentation, it was applied a rolling collisional energy with a collisional energy spread of 15 eV. 1 µg of peptides were loaded in the sample loop. The separation was carried out with a 10-cm capillary column at 1000 nl/min flow for a 20-min gradient from 5 to 30% of buffer B.

H) Short gradient-Method 5. 64 variably sized precursor co-isolation windows scheme. The SWATH windows cover the precursors ions in the range of 360-1460 m/z and 50-2000 in the MS2 SWATH scans,

the accumulation time was 250 ms for the MS1 and 50 ms for each SWATH window, resulting in a cycle time of 3.5 s. For fragmentation, it was applied a rolling collisional energy with a collisional energy spread of 15 eV. 2 µg of peptides were loaded in the sample loop. The separation was carried out with a 10-cm capillary column at 1000 nl/min flow for a 90-min gradient from 5 to 30% of buffer B.

Panel B. Pearson (upper triangle) and Spearman correlation (lower triangle) of fragment ions intensities across the tested conditions (90 min gradient vs. 20 min gradient).

Effect of the column length, flow rate, gradient length and number of DIA/SWATH variable windows in terms of the correlation of the total fragment intensities. The SWATH-MS data were analyzed using OpenSWATH, PyProphet and TRIC workflow (see Methods section). No further normalization was applied. From the plot results clear as the SWATH acquisition method [i.e. SWATH number of windows; Condition E (32 SWATH windows) vs Condition D (64 SWATH windows) and Condition F (100 SWATH windows)] influences most the performances of the analysis with respect that the column length (i.e. Condition A vs Condition B) and the flow rate (i.e. Conditions A/B vs Condition C).

Panel C. Number of peptides (left panel) and number of proteins (right panel) identified across the tested conditions.

Effect of the column length, flow rate, gradient length and number of DIA/SWATH variable windows in terms of total number of peptides and proteins identified.

The short gradient decreases the number of peptides identified by 53% (Condition A vs Condition D), whereas of 24% the total proteins compared to the long gradient. As shown for the correlation analysis, the major effect observed for short gradients is determined by the selection of the number of SWATH windows and the MS2 scan range. The 100 SWATH window method (Condition F) showed 76% and 53% of peptides and protein loss, respectively, with respect to the standard conditions (Condition A). The smaller MS2 scan range (Condition H) improved the performances of 24% and 13% of peptides and proteins characterized, respectively, in comparison with the broader MS2 scan range (Condition D).

A

Condition	N variable windows	MS^2 scan time (msec)	MS^2 mass range	Cycle time (msec)
A	32	40	350-1500	1430
B	32	40	50-1500	1430
C	32	40	50-2000	1430
D	64	20	350-1500	1430
E	64	20	50-1500	1430
F	64	20	50-2000	1430

B

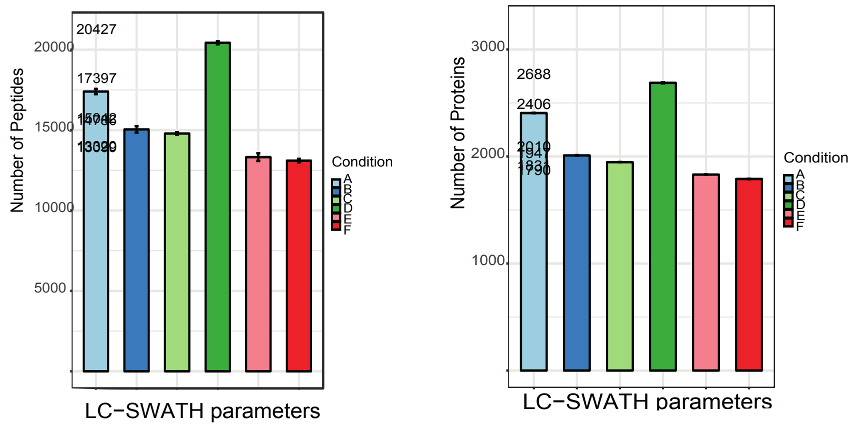

C

Condition	N variable windows	MS^2 scan time (msec)	MS^2 mass range	Cycle time (msec)
A	16	80	350-1500	1430
B	24	54	350-1500	1446
C	32	40	350-1500	1430
D	40	32	350-1500	1430
E	48	27	350-1500	1446
F	56	23	350-1500	1438
G	64	20	350-1500	1430

D

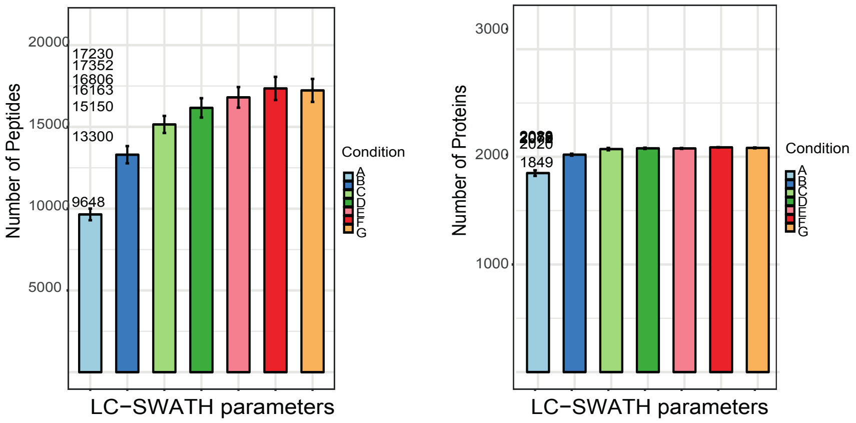

Supplementary Figure 2 - Effect of DIA/SWATH optimization on peptide detection rates

Effect of the MS2 scan range (Panels A and B) and number of SWATH windows (Panels C and D) in the DIA/SWATH performances (number of peptides and proteins identified). All the runs were performed on Evosep One system couple to an TripleTOF 5,600 instrument equipped with a NanoSpray III ion source selecting the "60 samples per day" method and using the nanoConnect LC column (8 cm column, ID 100 µm packed with 3 µm Reprosil, PepSep) (see Methods section).

Panel A. Short scheme of the different conditions tested for the MS2 scan range.

A) 32 variably sized precursor co-isolation windows. The SWATH windows cover the precursors ions in the range of 350-1460 m/z and 350-1500 in the MS2 SWATH scans, the accumulation time was 100 ms for the MS1 and 40 ms for each SWATH window, resulting in a cycle time of 1.430 s. For fragmentation, it was applied a rolling collisional energy with a collisional energy spread of 15 eV. 1 µg of peptides were loaded in the sample loop.

B) 32 variably sized precursor co-isolation windows with the MS2 SWATH scans range of 50-1500. The other parameters were the same of Condition A.

C) 32 variably sized precursor co-isolation windows with the MS2 SWATH scans range of 50-2000. The other parameters were the same of Condition A.

D) 64 variably sized precursor co-isolation windows. The SWATH windows cover the precursor ions in the range of 350-1460 m/z and 350-1500 in the MS2 SWATH scans, the accumulation time was 100 ms for the MS1 and 20 ms for each SWATH window, resulting in a cycle time of 1.430 s. For fragmentation, it was applied a rolling collisional energy with a collisional energy spread of 15 eV. 1 µg of peptides were loaded in the sample loop.

E) 64 variably sized precursor co-isolation windows with the MS2 SWATH scans range of 50-1500. The other parameters were the same of Condition D.

F) 64 variably sized precursor co-isolation windows with the MS2 SWATH scans range of 50-2000. The other parameters were the same of Condition D.

Panel B. Number of peptides (left panel) and number of proteins (right panel) identified across the conditions tested for the MS2 scan range.

Effect of the MS2 scan range in the DIA/SWATH performances in terms of number of peptides and proteins identified.

The smaller MS2 scan range improves the numbers of peptides and proteins identified both with 32 and 64 SWATH windows methods. For the 32 SWATH windows method, the 350-1500 MS2 scan range (Condition A) improves of ~14% and ~16% the number of peptides and proteins identifications (with respect of conditions B and C). For the 64 SWATH windows method, the 350-1500 MS2 scan range (ConditionD) improves of ~35% and ~32% the number of peptides and proteins identifications (with respect of conditions E and F). As shown here, the influence of the MS2 scan range in the mass spectrometry acquisition increases with an increased number of SWATH windows; a smaller mass range improves the sensitivity of the detection and allowing a higher number of events for the same full cycle time (1.430 secs).

Panel C. Short scheme of the different conditions tested for the number of SWATH windows.

A) 16 variably sized precursor co-isolation windows. The SWATH windows cover the precursor ions in the range of 350-1460 m/z and 350-1500 in the MS2 SWATH scans, the accumulation time was 100 ms for the MS1 and 80 ms for each SWATH window, resulting in a cycle time of 1.430 s. For fragmentation, it was applied a rolling collisional energy with a collisional energy spread of 15 eV. 1 µg of peptides were loaded in the sample loop.

B) 24 variably sized precursor co-isolation windows. The accumulation time for each SWATH window is 54 ms, for a total cycle time of 1.446 s. The other parameters were the same of Condition A.

C) 32 variably sized precursor co-isolation windows. The accumulation time for each SWATH window is 40 ms, for a total cycle time of 1.430 s. The other parameters were the same of Condition A.

D) 40 variably sized precursor co-isolation windows. The accumulation time for each SWATH window is 32 ms, for a total cycle time of 1.430 s. The other parameters were the same of Condition A.

E) 48 variably sized precursor co-isolation windows. The accumulation time for each SWATH window is 27 ms, for a total cycle time of 1.446 s. The other parameters were the same of Condition A.

F) 56 variably sized precursor co-isolation windows. The accumulation time for each SWATH window is 23 ms, for a total cycle time of 1.438 s. The other parameters were the same of Condition A.
G) 64 variably sized precursor co-isolation windows. The accumulation time for each SWATH window is 20 ms, for a total cycle time of 1.436 s. The other parameters were the same of Condition A.

Panel D. Number of peptides (left panel) and number of proteins (right panel) identified across the conditions tested for the number of SWATH windows. Evaluation of the performances of different DIA/SWATH methods in function of the number of variable SWATH windows. The shorter gradient
The smaller MS2 scan range improves the numbers of peptides and proteins identified both with 32 and 64 SWATH windows methods. For the 32 SWATH windows method, the 350-1500 MS2 scan range (Condition A) improves of ~14% and ~16% the number of peptides and proteins identifications (with respect of conditions B and C). For the 64 SWATH windows method, the 350-1500 MS2 scan range (ConditionD) improves of ~35% and ~32% the number of peptides and proteins identifications (with respect of conditions E and F). As shown here, the influence of the MS2 scan range in the mass spectrometry acquisition increases with an increased number of SWATH windows; a smaller mass range improves the sensitivity of the detection and allowing a higher number of events for the same full cycle time (1.430 secs).

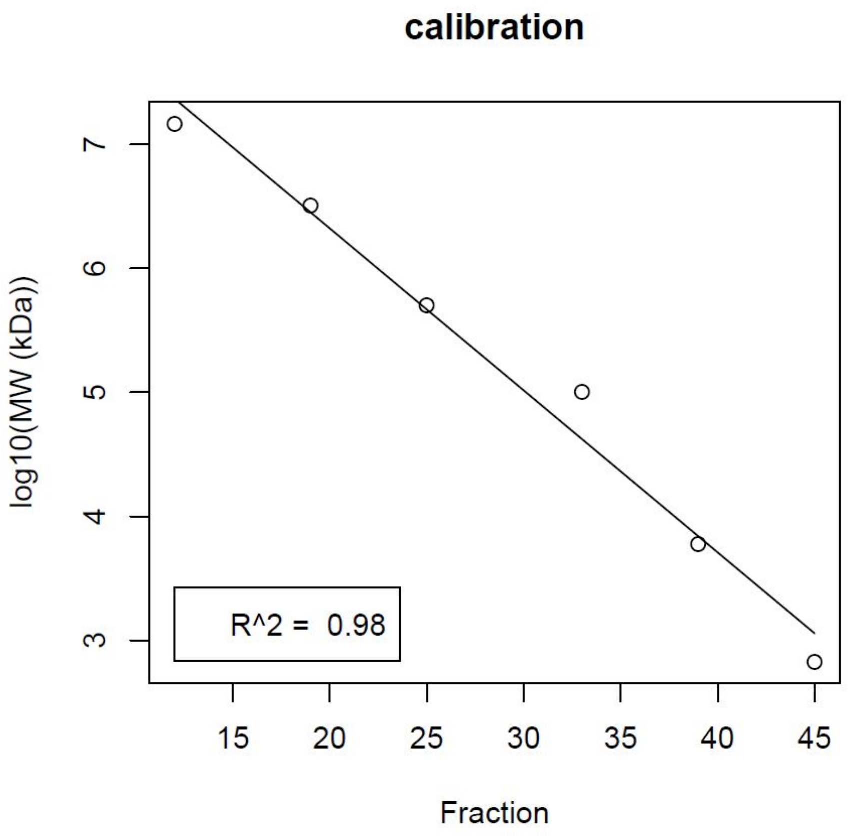

Supplementary figure 3 – SEC molecular weight calibration

Example of a molecular weight calibration based on the log-linear relationship of the calibrant's molecular weights and the elution fraction in the SEC separation.

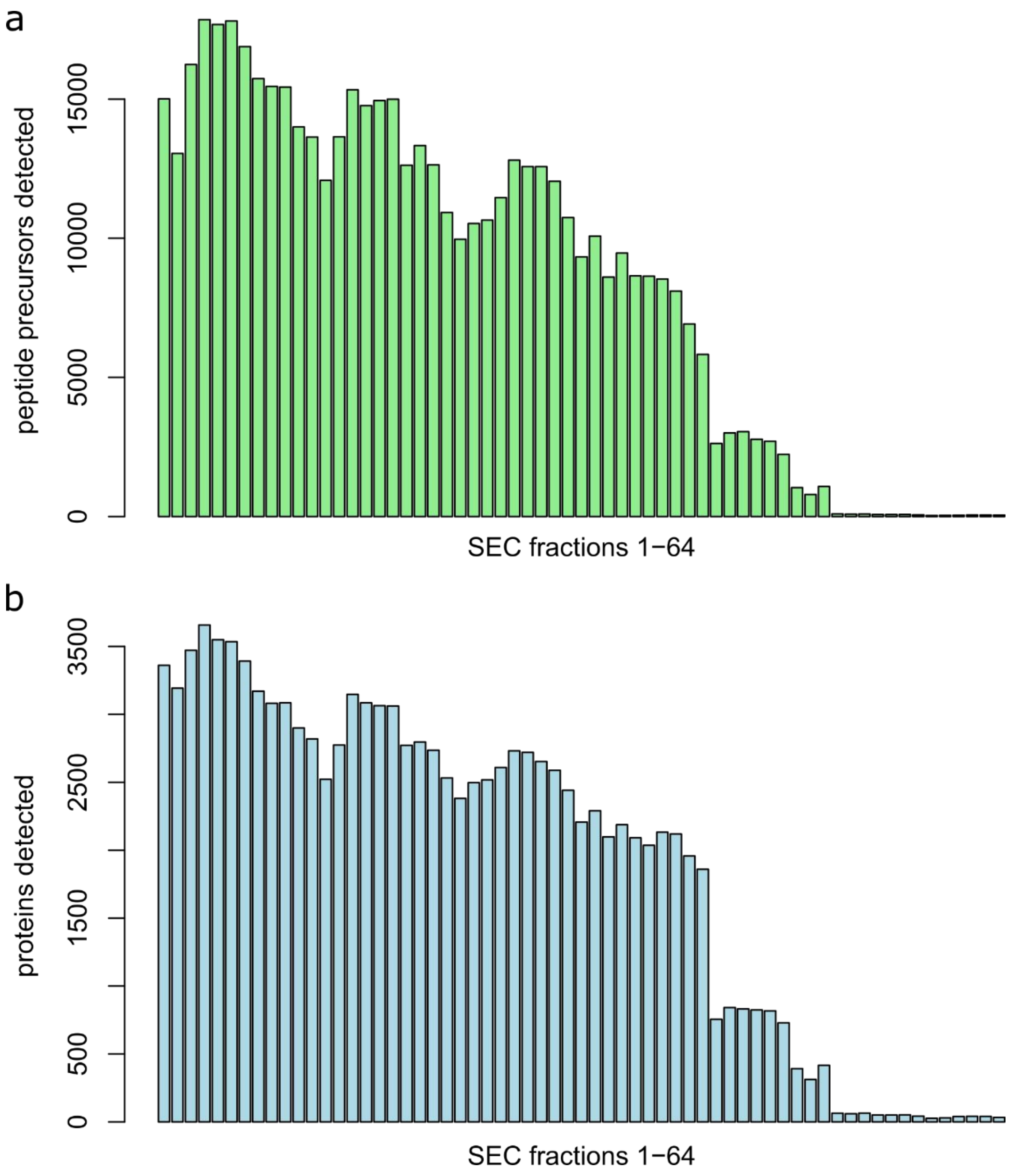

Supplementary figure 4 – Peptide precursor and protein numbers detected by SEC fraction

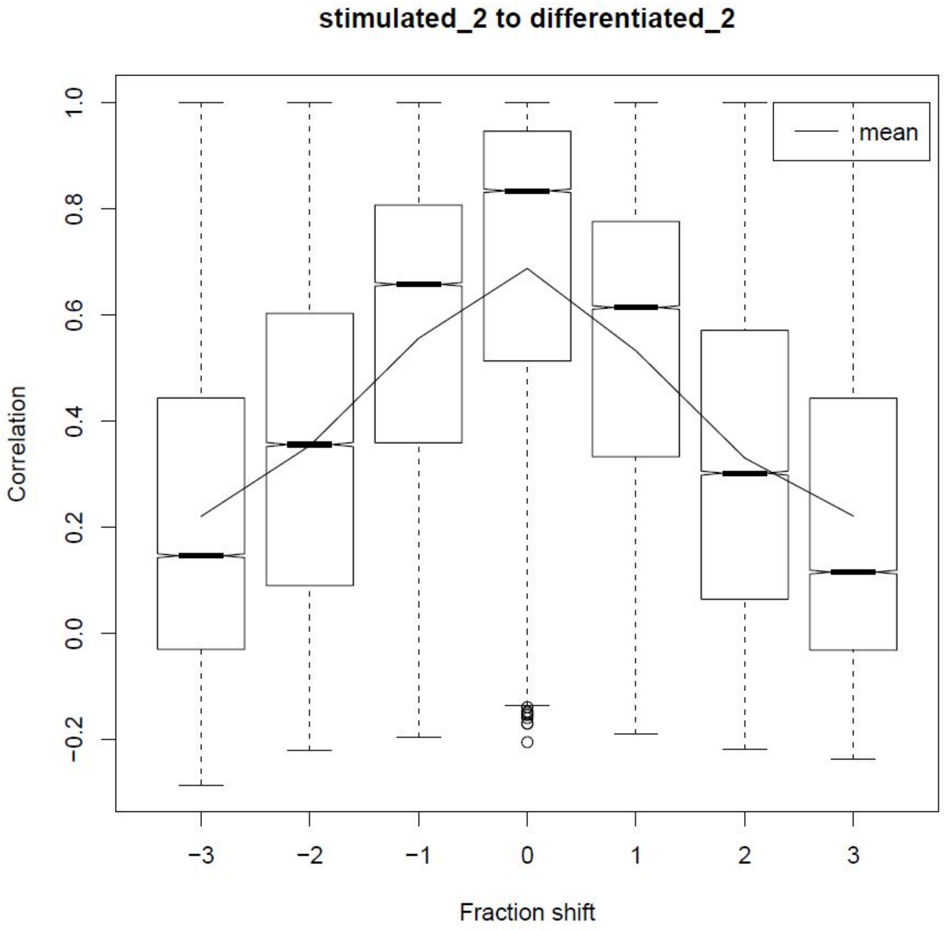

Supplementary figure 5 – Pairwise SEC alignment

Representative example of a pair-wise alignment between the peptide-level SEC traces to illustrate different fraction shift offsets. To determine whether 2 SEC runs need to be aligned we compute the correlation coefficients all peptides over all SEC fractions, and further calculate this correlation when the fractions are offset between runs by a number of fractions (-3 to 3). Where the maximum correlation is at 0 offset there is no requirement to perform any correction.

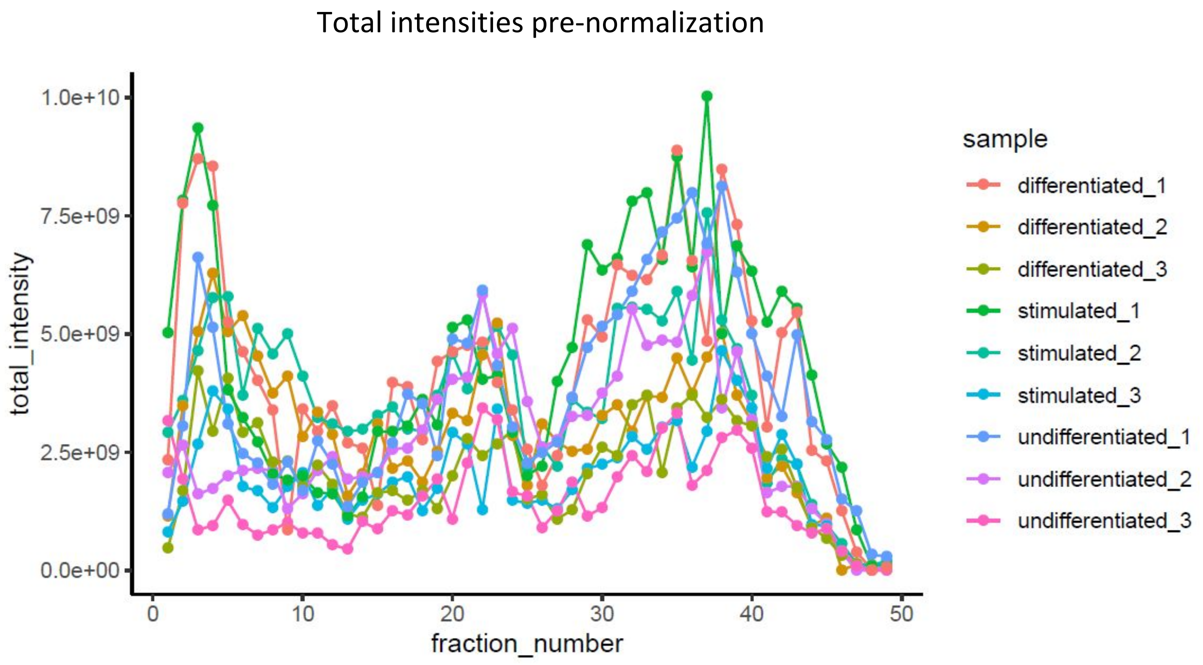

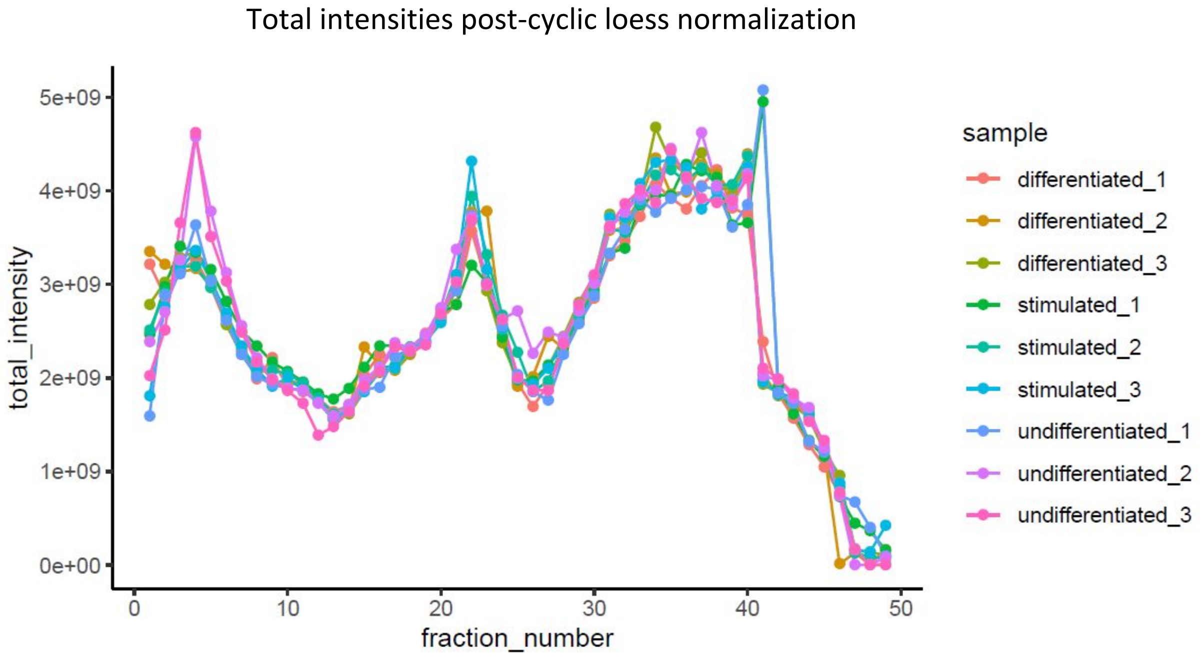

Supplementary figure 6 – Cyclic loess normalization

Collective total ion intensities for all 9 SEC runs before and after cyclic loess normalization for all 9 runs.

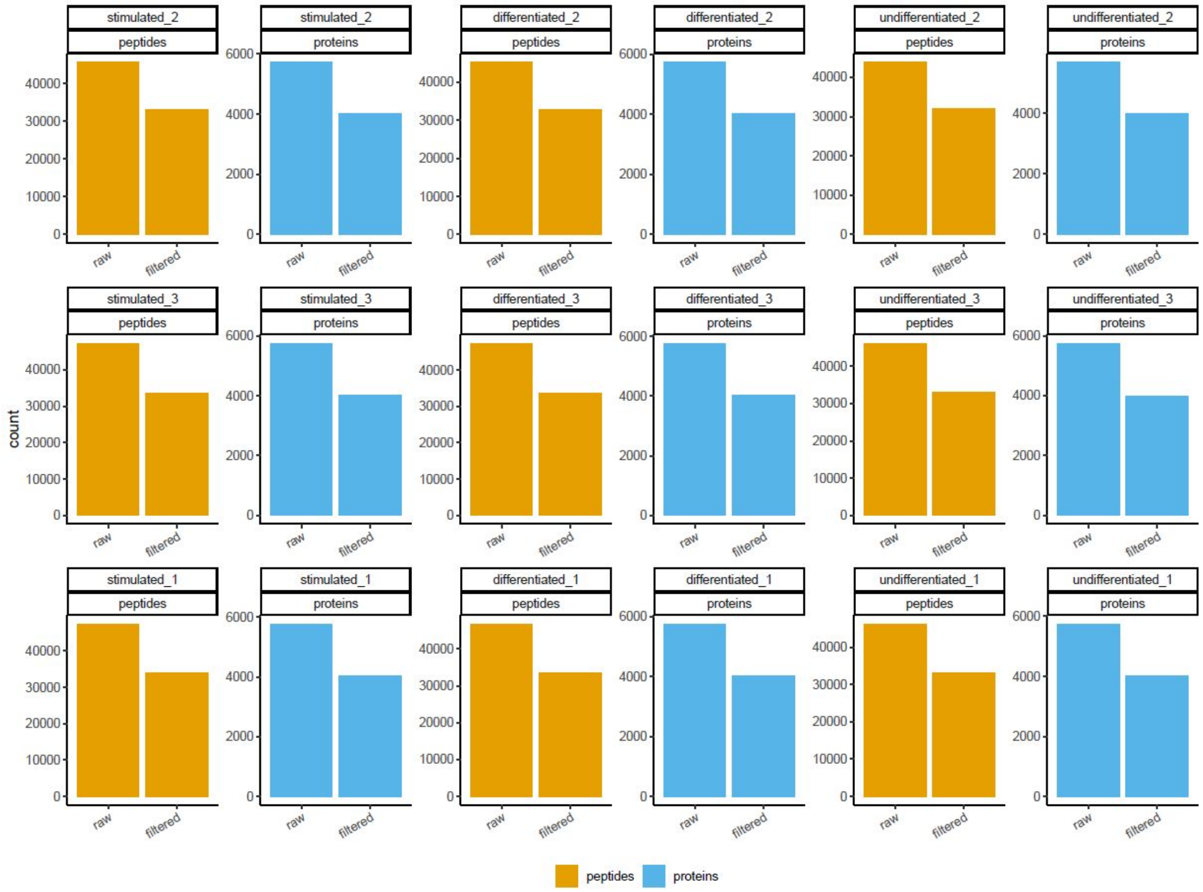

Supplementary figure 7 – Peptide detection rates and effect of filtering

Number of peptides (orange) or proteins (in blue) before and after the 3 peptide-filtering steps. The filtering steps are based on the identification of peptides in consecutive fractions and based on the correlation of sibling peptides mapping to the same proteins.

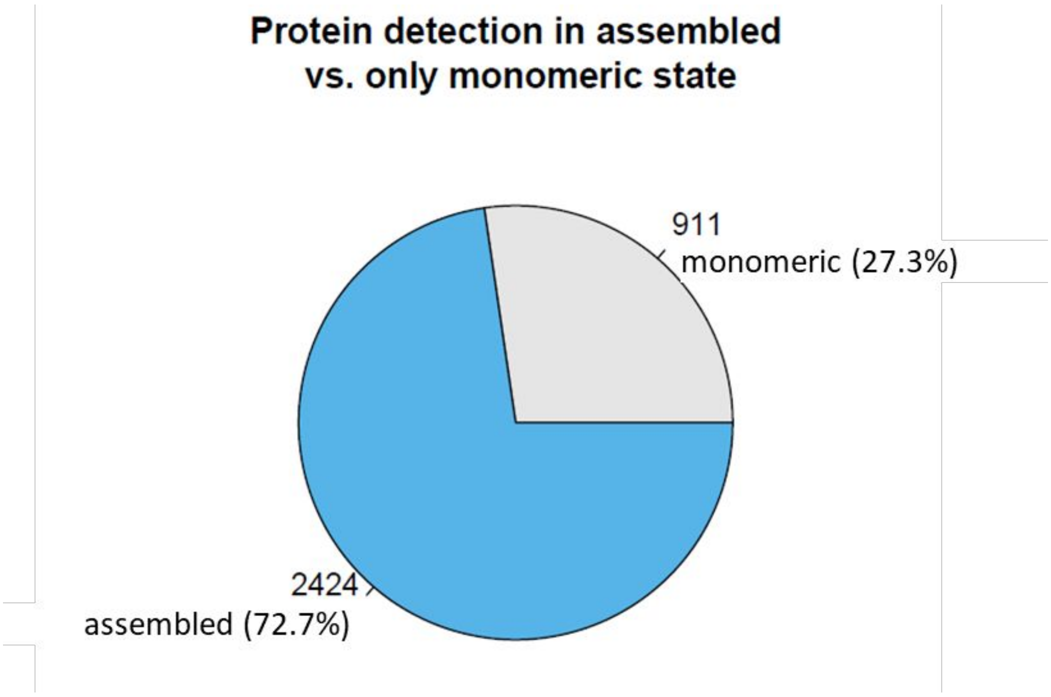

Supplementary figure 8 – Proteins detected in assembled vs. monomeric state
The number of proteins for which at least one feature was detected in an 'assembled' state (> 2 x monomer molecular weight) versus those detected only in the 'monomeric' state (< 2 x monomeric molecular weight) are depicted.

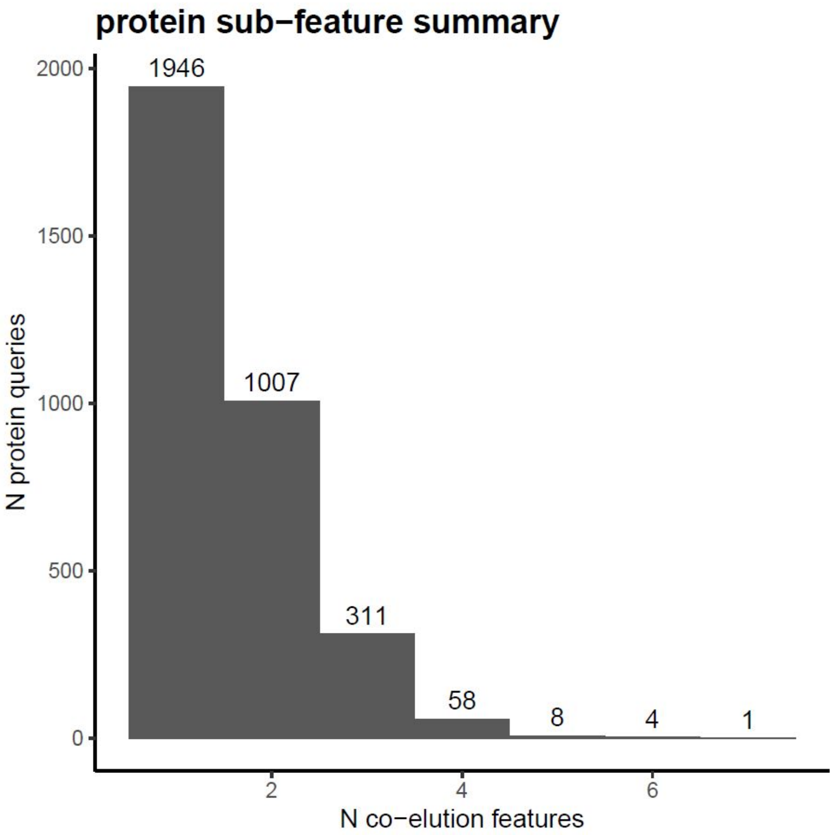

Supplementary figure 9 – Detected protein feature summary

The number of SEC elution features a given protein was detected is summarized for all detected proteins after filtering.

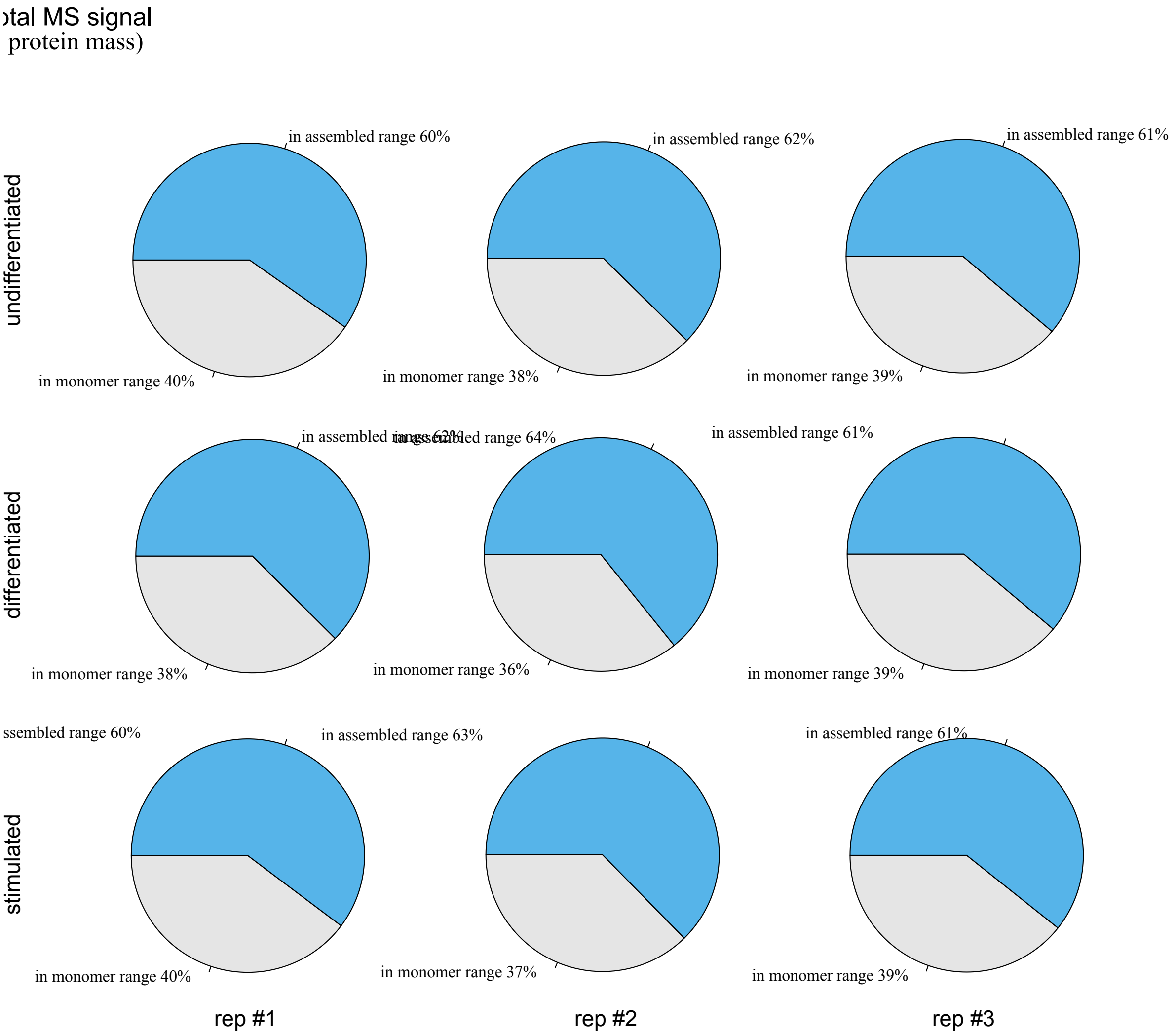

Supplementary figure 10 – Protein mass in assembled state

The fraction of protein mass as estimated by the MS signal intensity to be in an assembled (> 2 x monomer molecular weight -- blue) versus monomeric (< 2 x monomer molecular weight -- grey) state is shown.

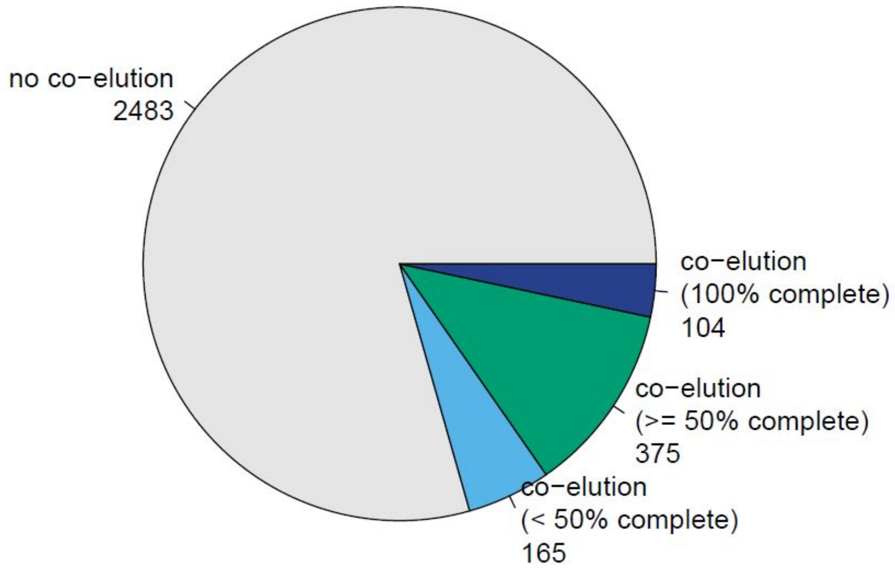

Supplementary figure 11 – Protein complex detection rates as a fraction of complex hypotheses
The number and fraction of protein complexes detected in THP1 at 5% FDR at the complex-detection level from the merged CORUM and String (clustered) hypothesis set are shown.

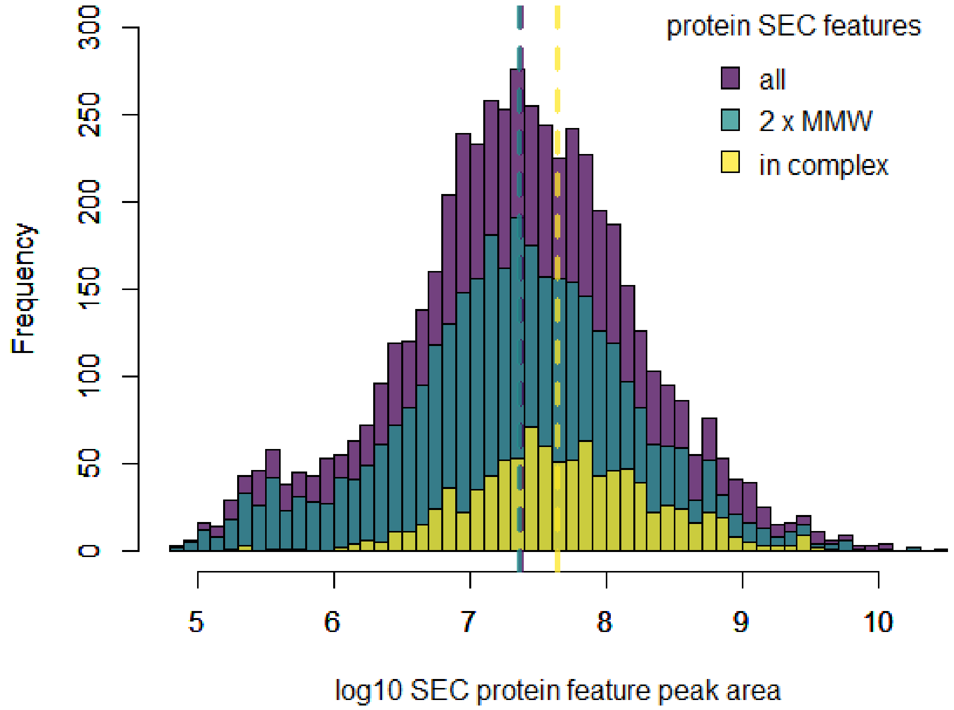

Supplementary figure 12 – Distribution of SEC protein features dependent on MW and detection in complex-centric analysis

The distribution of all SEC protein features (purple), the subset of those features that appear at >2x the expected monomer weight and are expected to be in complex (green), and those that are detected as a subunit of protein complex in the complex-centric analysis (yellow) are shown. Matching of protein SEC features to protein complexes is anti-conservative using an apex fraction tolerance of ±5 SEC fractions.
